# Acemannan Induced Bone Regeneration in Lateral Sinus Augmentation Based on Cone Beam Computed Tomographic and Histopathological Evaluation

**DOI:** 10.1155/2020/1675653

**Published:** 2020-02-13

**Authors:** Hai Anh Trinh, Van Viet Dam, Wijit Banlunara, Polkit Sangvanich, Pasutha Thunyakitpisal

**Affiliations:** ^1^Multidisciplinary Dental Biomaterials Science Program, Graduate School, Chulalongkorn University, Bangkok 10330, Thailand; ^2^Department of Implantology, Hanoi National Hospital of Odonto-Stomatology, Hanoi 100000, Vietnam; ^3^Department of Pathology, Faculty of Veterinary Science, Chulalongkorn University, Bangkok 10330, Thailand; ^4^Department of Chemistry, Faculty of Science, Chulalongkorn University, Bangkok 10330, Thailand; ^5^Department of Anatomy, Multidisciplinary Dental Biomaterials Science Program, Research Unit of Herbal Medicine, Biomaterial and Material for Dental Treatment, Faculty of Dentistry, Chulalongkorn University, Bangkok 10330, Thailand

## Abstract

Acemannan, the major polysaccharide extracted from Aloe vera, is biomaterial that has demonstrated osteoinductive effects *in vitro* and *in vivo.* However, the effect of acemannan sponges on bone formation in open-type sinus augmentation has not evaluated. Here, we report a case study using radiographic and histological analyses to investigate the effect of acemannan on bone formation after lateral sinus lift surgery. The case was a 57-year-old female patient with an atrophic left posterior maxilla who underwent lateral sinus lift using an acemannan sponge using the two-stage procedure. In the first stage, an acemannan sponge was inserted through the bony window and placed between the antral floor and the elevated sinus membrane. Cone beam computed tomography (CBCT) images were taken immediately as baseline and 6-month postoperation for evaluation. A bone core specimen was also obtained for histological examination at the time of implant placement. The histological results revealed new bone formation, and the CBCT images demonstrated increased alveolar bone height at 6-month postoperation. Our findings suggest that an acemannan sponge could be a biomaterial for inducing bone formation in sinus lift surgery.

## 1. Introduction

Limited residual alveolar bone height is frequently encountered in the edentulous posterior maxilla due to its anatomy and physiology, sinus enlargement, and postextraction alveolar bone resorption [1]. These circumstances result in thin antral floor bone, which is unfavorable for dental implant placement. Thus, various bone augmentation techniques have been introduced to overcome this problem. A lateral sinus lift is the recommended technique when the residual bone height is less than 5 mm, which is performed by creating a bony window in the lateral wall of the maxillary sinus, followed by lifting the sinus membrane and placing a grafting material to enhance bone formation [2, 3]. This procedure can be performed as a one- or two-stage procedure. In cases of extremely minimal residual bone (1–3 mm), it may be difficult to achieve implant stability; in these cases, a two-stage procedure is performed [4]. The sinus bone is augmented at the first operation. The implant is then placed at the second operation at 6-month postoperation after the bone healing period.

Many bone substitute materials have been used for sinus bone augmentation including autografts, allografts, xenografts, and alloplasts [5]. Although an autograft is considered the optimum material for a bone graft due to its osteoconductive, osteoinductive, and osteogenic properties, donor site morbidity and limited quantities are disadvantages of this material [6]. In addition, allografts and xenografts have a risk of disease transmission and religious concerns [7]. Many researchers have been searching for alternative biomaterials to overcome these drawbacks.

*Aloe vera* is known for its wound healing effect. Acemannan, the major polysaccharide fraction from Aloe vera, has been reported as a promising biomaterial for bone regeneration [8]. Acemannan has shown osteoinductive effects, enhancing the alveolar bone and bone density in tooth extraction sockets [9, 10]. However, the radiographic and histological evaluation of acemannan on bone formation in direct sinus lift surgery has not been investigated. In this study, acemannan was extracted from fresh Aloe vera pulp gel and characterized with ^13^C- and ^1^H-nuclear magnetic resonance spectroscopy and Fourier transform infrared spectroscopy as previously described [11, 12]. The acemannan sponge was prepared by dissolved 150 mg acemannan in 3 ml sterile distilled water, frozen at -80°C overnight, and then lyophilized for 24 h [10]. The sponge was sterilized by gamma irradiation (Thailand Institute of Nuclear Technology, Bangkok, Thailand) and stored in a desiccator until used. In this case, the patient underwent lateral sinus augmentation with an acemannan sponge at the first stage without immediate implant placement. The outcome was evaluated radiographically and histologically.

## 2. Case Presentation

**Figure 1:**
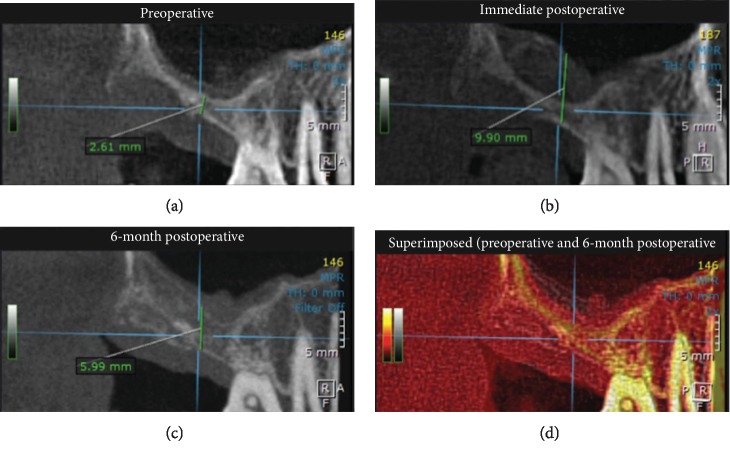
The sagittal view CBCT images show the left posterior edentulous maxillary region of the patient at preoperation (a), immediate postoperation (b), 6-month postoperation (c), and superimposed CBCT image between preoperation and 6-month postoperation of the lateral sinus augmentation (d).

A 57-year-old, nonsmoking female patient with good general health was referred to the Department of Implantology, National Hospital of Odonto-Stomatology, Hanoi, Vietnam, with a complaint of loss of chewing ability on the upper left side. Her clinical examination found that the upper left second and third molars were missing. The patient's dental history revealed that the upper left second and third molars were extracted due to severe aggressive periodontal disease many years ago, and the patient refused to use a removable denture. The initial CBCT evaluation demonstrated left maxillary sinus pneumatization and a 2.61 mm residual bone height in the edentulous region underneath the sinus (Figure 1(a)). Based on these findings, a two-stage lateral sinus lift procedure was planned. Bleeding time, coagulation time, and clot retraction time assays were performed. The patient was informed about the study and procedure protocol and agreed to participate in this study.

The patient was prescribed 600 mg clindamycin 1 h before surgery [13] and rinsed with 0.12% chlorhexidine solution (Peridex 3M ESPE, 3M Dental Products, St. Paul, MN, USA) for 1 min prior to the operation. Local anesthesia (2% lidocaine with epinephrine 1 : 80,000, Lignospan Special, Septodont, Saint-Maur-des-Fosses, France) was administered. Subsequently, a midcrestal incision and releasing incisions were performed, and a full-thickness mucoperiosteal buccal flap was raised to expose the alveolar bone. A bony window in the lateral wall of the sinus was created using an SLA KIT (Neobiotech, Seoul, South Korea). The lateral bony window wall was removed with a periosteal elevator, and the sinus membrane was lifted. A 150 mg acemannan sponge was placed on the antral floor under the elevated sinus membrane. The bony window wall was repositioned, and the incisions were sutured using a 4.0 nonabsorbable suture (Ethicon, Inc., Somerville, NJ, USA). A CBCT scan was immediately performed as baseline.

Postoperatively, the patient was prescribed 300 mg clindamycin 3 times daily for 7 days, 0.12% chlorhexidine solution for mouthwash (10 ml for 1 min, 3 times/day for 10 days), and a nonsteroidal analgesic (ibuprofen 400 mg, 3 times/day for 3 days; as needed) [14]. The patient was called to ask about any postoperative complications at days 1 and 3. The patient was seen 7 days postoperatively for suture removal and clinical evaluation. The patient was then recalled at 6-month postoperation to assess bone formation using CBCT. Implant placement was performed at the second stage. A sinus core biopsy specimen was obtained at the implant site prior to implant placement using a 3 mm trephine bur and stored in Bouin's fixative. The bone core sample was submitted for histopathological examination. During the study, the patient was instructed not to wear any denture to prevent interference with wound healing.

### 2.1. Radiographic Image Acquisition

The CBCT radiograph was performed immediately and 6-month postoperation using a Planmeca ProMax 3D (Planmeca, Helsinki, Finland) with fixed exposure parameters of 96 kV, 7 mA, 50 × 55 mm FOV, 75 *μ*m slice thickness, and 15.152 seconds. The images were obtained to evaluate the residual bone quality and quantity and any possible maxillary sinus pathology. To minimize the error from radiographic image alignment, the OnDemand3D™ fusion module software (Cybermed Inc., Seoul, South Korea) was used to generate a superimposed image in the sagittal plane. With the advancement of this technology, the difference between pre- and postoperative images could be accurately and reliably observed in the superimposed image to evaluate the treatment results [15]. The method uses a basic concept from information theory and mutual information (MI) as a matching criterion [16]. To perform the image fusion process, the manual registration was used first to roughly align the image datasets. Next, an automated registration tool was used to automatically fuse the image datasets. The residual bone heights between baseline and 6-month postoperation were determined and compared. The residual bone heights were measured at the site where the implant would be placed.

### 2.2. Histopathological Processing

The bone core specimen was demineralized in 10% formic acid, dehydrated in ethanol–acetone, and embedded in paraffin. 6 *μ*m thickness sections were prepared in the longitudinal midline direction and stained with hematoxylin-eosin (H&E). The sections were scanned, recorded by slide digitalization (Pannoramic Scan, 3DHistech Ltd., Budapest, Hungary), and analyzed using the CaseViewer 2.0 (3DHistech Ltd., Budapest, Hungary) program. The sections were examined by a blinded pathologist. The results are presented descriptively.

### 2.3. Clinical and CBCT Evaluation

There were no adverse events during the surgical procedure or healing period. The patient did not experience any postsurgical complications. The immediate postoperative CBCT images showed that the sinus wall membrane was elevated and the space was filled by the acemannan sponge (Figure 1(b)). The 6-month postoperative CBCT images revealed that the radiographic bone height had increased from 2.61 to 5.99 mm (Figures 1(c) and 1(d)).

### 2.4. Histopathological Evaluation

**Figure 2:**
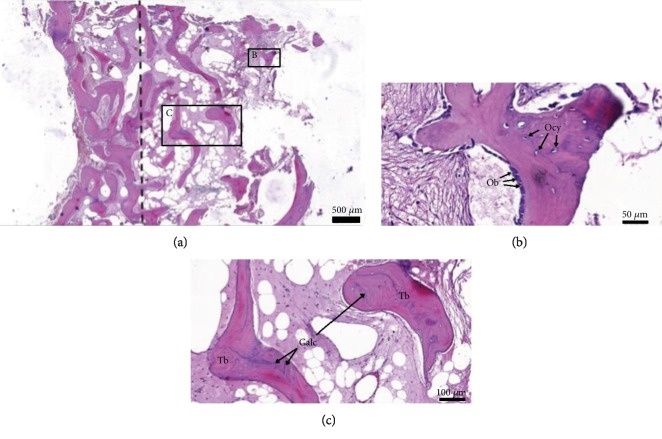
(a) Low-magnification image of the histopathological evaluation using H&E staining of the 6-month postoperation bone core specimen. The dashed line represents the border between the pre- and postoperation residual bone. (b) High-magnification image demonstrates active cuboidal osteoblasts (Ob) and osteocytes (Ocy) in lacunae. (c) High-magnification image shows thick trabeculae (Tb), bone lacunae, and calcification zone (Calc).

The sections did not demonstrate any signs of inflammation. New bone formation was observed. Active osteoblasts were present on the periphery of the newly formed bone. Numerous osteocytes in lacunae illustrated the vitality of the bone specimen (Figures 2(a) and 2(b)). A zone of calcification confirmed the mineralization process. Thick bone trabeculae with lamellae were noted (Figure 2(c)).

## 3. Discussion

The use of allografts, xenografts, and alloplasts has been recommended to increase bone height in sinus augmentation procedures, rather than using an autograft. Because the radiodensity of a bone graft is similar to that of a native bone, the quality of the increased bone height as a living hard tissue for a sustainable outcome has been questioned.

In the present study, the CBCT images showed alveolar bone height increased approximately 2-fold from the baseline height at 6-month postoperation. The histological images confirmed the new bone formation with many osteoblasts and osteocytes present. Bone trabeculae with lamella and calcification zones were also observed. In addition, no inflammatory response or tissue reaction was observed. The sponge was resorbed and replaced with new bone. This finding indicated that the acemannan sponge was biocompatible with the maxillary sinus. Taken together, our results demonstrated the osteoinductive property of the acemannan sponge. These results correspond with our report that demonstrated using acemannan in indirect sinus augmentation and simultaneous implant placement increased bone formation [17].

Based on the study's limitations, a precise explanation of the underlying mechanism of acemannan on osseous regeneration could not be determined. A possible explanation is that, as a 3-dimensional interconnected porous scaffold, the acemannan sponge absorbs blood and/or serum from the surrounding tissue to stabilize the blood clot to maintain its volume for bone growth [9]. Acemannan may increase bone formation by increasing cell proliferation, osteoblast differentiation, growth factor and extracellular matrix secretion, and mineralization [8, 9]. Moreover, the anti-inflammatory and immunomodulatory effects of acemannan have been reported [18].

This polysaccharide will facilitate the inflammatory reaction through macrophage activation and the release of cytokines and growth factors. Interleukins (ILs), tumor necrosis factor alpha (TNF-*α*), transforming growth factor beta (TGF-*β*), platelet-derived growth factor (PDGF), endothelial growth factor (EGF), bone morphogenetic proteins (BMPs), and vascular endothelial growth factor (VEGF) have been associated with bone repair and regeneration [19, 20].

It should be noted that the increased bone height in this study is limited. The postoperative bone height gain was approximately 6 mm from the 3 mm initial bone height; however, the minimum implant length for predictable success is 10 mm [21]. Therefore, adding acemannan as a biomolecule in an osteoconductive scaffold (such as xenograft, allograft, or synthetic calcium phosphate) in sinus augmentation and in implant placement in the esthetic zone should be investigated.

## 4. Conclusion

The safety and biocompatibility of the acemannan sponge with the maxillary sinus were investigated. The osteoinductive property of acemannan in a sinus lift procedure was demonstrated using CBCT and histological analysis. Further studies with a larger sample size should be performed.
